# Atherogenic Index Reduction and Weight Loss in Metabolic Syndrome Patients Treated with A Novel Pectin-Enriched Formulation of Bergamot Polyphenols

**DOI:** 10.3390/nu11061271

**Published:** 2019-06-04

**Authors:** Antonio Soccorso Capomolla, Elzbieta Janda, Sara Paone, Maddalena Parafati, Tomasz Sawicki, Rocco Mollace, Salvatore Ragusa, Vincenzo Mollace

**Affiliations:** 1 Villa dei Gerani Hospital, 89900 Vibo Valencia, Italy; scapomolla@gmail.com; 2 Department of Health Sciences, Magna Graecia University, Campus Germaneto, 88100 Catanzaro, Italy; mparafati@unicz.it (M.P.); t.sawicki@pan.olsztyn.pl (T.S.); rocco.mollace@gmail.com (R.M.); sragusa@unicz.it (S.R.); 3 Interregional Research Center for Food Safety and Health, 88100 Catanzaro, Italy; sara.paone06@gmail.com; 4 Institute of Animal Reproduction and Food Research of the Polish Academy of Sciences, 10-748 Olsztyn, Poland; 5 San Raffaele, Istituto di Ricovero e Cura a Carattere Scientifico (IRCCS), Pisana, 00163 Rome, Italy

**Keywords:** atherosclerosis, metabolic syndrome, insulin resistance, bergamot polyphenol fraction (BPF), body mass index, clinical trial, cardiovascular risk factors, obesity

## Abstract

Bergamot flavonoids counteract dyslipidemia and hyperglycemia but fail to induce a significant weight loss. Here, we evaluated the efficacy of bergamot polyphenol extract complex (BPE-C), a novel bergamot juice-derived formulation enriched with flavonoids and pectins, on several metabolic syndrome parameters. Obese patients with atherogenic index of plasma (AIP) over 0.34 and mild hyperglycemia were recruited to a double-blind randomized trial comparing two doses of BPE-C (650 and 1300 mg daily) with placebo. Fifty-two subjects met the inclusion criteria and were assigned to three experimental groups. Fifteen subjects per group completed 90 days-trial. BPE-C reduced significantly fasting glucose by 18.1%, triglycerides by 32% and cholesterol parameters by up to 41.4%, leading to a powerful reduction of AIP (below 0.2) in the high dose group. The homeostasis model assessment of insulin resistance (HOMA-IR) and insulin levels were also reduced. Moreover, BPE-C decreased body weight by 14.8% and body mass index by 15.9% in BPE-C high group. This correlated with a significant reduction of circulating hormones balancing caloric intake, including leptin, ghrelin and upregulation of adiponectin. All effects showed a dose-dependent tendency. This study suggests that food supplements, containing full spectrum of bergamot juice components, such as BPE-C efficiently induce a combination of weight loss and insulin sensitivity effects together with a robust reduction of atherosclerosis risk.

## 1. Introduction

Metabolic syndrome (MetS) is a cluster of several cardiometabolic risk factors, including hyperglycemia or glucose intolerance, high levels of triglycerides (TG) and low-density lipoprotein cholesterol (LDL-C) and low levels of high-density lipoprotein cholesterol (HDL-C), hypertension, abdominal adiposity and obesity [1,2]. It is diagnosed based on the presence of at least three metabolic alterations listed above, but insulin resistance and visceral adiposity are the most common features of MetS pathophysiology [3]. MetS prevalence ranges from 10% up to 85% of general population worldwide and is higher in industrialized countries. In Europe, the mean MetS incidence is 24.3% and it increases with advancing age (from 3.7 in the group aged 20–29 years to more than 30% in the subjects 70 years and older) [3]. Around 20–25% of Italians have MetS and reflect the European mean [4]. The treatment of MetS is the main strategy to prevent cardiovascular complications, such as atherogenesis [1]. Atherosclerotic lesions can form in the presence of an unfavorable plasma lipid profile that can be characterized by atherogenic index of plasma (AIP). It is defined as logarithm [log] of the ratio of TG to HDL-C plasma concentrations and thus it depends mainly on circulating fat levels. AIP values over 0.21 indicate high risk of atherosclerosis and inversely correlate with cardiovascular health [5,6]. AIP can be also considered as a strong and independent predictor for coronary artery disease [5,7,8]. 

The energy homeostasis and, in particular body fat and food intake, are altered in MetS and this is associated with abnormalities in circulating hormones with a pivotal role in the regulation of energy balance in the body, such as adiponectin, leptin, and ghrelin. In fact, both the levels of these hormones, as well their ratio are known to be altered in MetS patients [9]. Adiponectin is considered cardioprotective since it improves lipid and glucose metabolism, increases insulin sensitivity [9]. Several observational studies have reported an inverse association between adiponectin serum levels and body weight (BW), total cholesterol (TotChol), TG levels, blood pressure, and insulin resistance, and a positive association with HDL-C levels [10]. On the contrary, there is a positive correlation of MetS parameters, such as insulin resistance and adiposity with plasma concentrations of leptin [11]. In fact, elevated plasma leptin is considered as an independent cardiovascular risk factor [12]. Ghrelin is a meal-initiating, acylated peptide secreted primarily by the stomach an endogenous ligand for the growth hormone receptor, which rapidly stimulates food intake when exogenously administered [13]. Plasma ghrelin concentrations correlate with appetite and food intake [14]. 

Overwhelming evidence suggests that many of the features of metabolic syndrome, including hormonal imbalance can be efficiently treated with natural approaches, such as fiber and polyphenol-rich diet and polyphenol-rich food supplements [15,16]. One of the most promising and effective food supplements used for the management of metabolic syndrome is Bergamot Polyphenol Fraction (BPF). BPF is a yellow powder extracted from bergamot (*Citrus bergamia* Risso et Poiteu) fruits, containing high levels of flavonoids, including 38 ± 2% of flavanones such as naringin, neoericitrin, neohesperidin, bruteridin and melitidin [17,18]. BPF was shown to counteract several features of MetS. In particular, TG and TotChol levels were strongly reduced, both alone as well as in combination with statins [19,20,21]. In line with this property, BPF has a particularly strong effect on lipid metabolism in the liver [17,22]. Accordingly, it prevents non-alcoholic fatty liver disease (NAFLD) induced by cafeteria diet (CAF) in rats [22], and in the same animal model boosts the therapeutic effect of the normocaloric diet on CAF-induced non-alcoholic steato-hepatitis (NASH) [17]. There is also an evidence indicating that BPF attenuates NAFLD and NASH in patients [23,24]. Hepatoprotective effects of BPF are associated with its ability to induce autophagy [18,22,25]. The proautophagic activity of BPF suggests that it may stimulate energy expenditure and prevent weight gain. Yet, little evidence has been collected on weight-loss effects of BPF.

In addition, the experimental data suggest that non-flavonoid components of fruits, including pectins, as well as other yet unidentified compounds, may enhance the pharmacological responses to flavonoid phytocomplex [26,27]. This may depend on microbiota-mediated or microbiota-independent mechanisms [28,29].

For this reason, we decided to evaluate the effect of the exsiccated bergamot juice extract containing 8% pectins, 8% vitamin C, enriched with BPF to increase the flavonoid content in a small-scale clinical trial on MetS patients with moderate hyperglycemia. The first aim of the study was to evaluate changes in serum lipid and glucose contents after 90 days treatment with two doses of BPE-C. The secondary aim was to evaluate a possible BW loss elicited by BPE-C treatment as well as its impact on serum levels of adiponectin, leptin and ghrelin in the same group of patients within the 90 days study period. 

## 2. Materials and Methods 

### 2.1. Food Supplement Used in The Study

BPE-C also known with a proprietary name as BΠE-complex^TM^ was developed and kindly provided by Herbal and Antioxidant Derivatives (H&AD) S.r.l. (Bianco, RC, Italy). BPE-C contains around 80% of BPE, which is a bergamot juice extract characterized by a reduced amount of carbohydrates and citric acid and around 20% of BPF^TM^ containing mainly bergamot juice flavonoids. The latter is added to increase the amount of five main bergamot flavanones (neoeriocitrin, naringin, neohesperidin, melitidin, bruteridin) to 38 ± 2%. BPE was obtained from a fresh bergamot juice by a partial pulp and citric acid removal and essential oil evaporation followed by an enzymatic reduction of carbohydrate content and a subsequent spray drying according to a proprietary procedure developed by H&AD. BPE contains >8% pectins and >8% ascorbic acid (vitamin C) and appears as a yellow powder. BPF^TM^ was obtained from a clarified bergamot juice according to a patented procedure (No. EP 2 364 158 B1) described also in Mollace et al. [20] Polyphenol content was routinely determined by high-pressure liquid chromatography [18,20]. 

### 2.2. Subjects

Participants, males and females between 40 to 80 years old, were recruited from MetS patients of San Raffaele IRCCS, Pisana, Rome, Italy and Villa dei Gerani Hospital, Vibo Valencia, Italy, who were not originally receiving lipid-lowering therapy. Inclusion criteria were: obesity with BMI >26, high TG >200 mg/dL, high TotChol >200 mg/dL, moderate glycemia: 130 >Glu > 100 mg/dL. 

Exclusion criteria were gastritis, presence of other diagnosed malignancies, pregnancy or breast-feeding, lack of compliance defined as not using the correct BPE-C dose or placebo for >1 week), and inability to give informed consent. The study protocol was given approval by the Institutional Ethics Committee and written informed consent was obtained from participants. This trial was registered at the *Magna Graecia* University of Catanzaro (UNICZ) Clinical Trials Registry (http://www.unicz.it/.../) under Trial No. 182/2016.

### 2.3. Study Design

This study was designed as a randomized, double-blind, placebo-controlled trial. Patients who met the inclusion criteria were assigned to either BPE-C low, n = 17 (one capsule = 650 mg BPE-C) or BPE high, *n* = 18, (2 capsules = 1300 mg BPE-C) treatment groups, or a matched placebo group (*n* = 17) for a period of 90 days. Placebo capsules contained the same amount of maltodextrin. BPE-C dose (650 mg once or twice daily) was established based on preliminary observations and the use of the same dose in our previous trial in MetS individuals [20]. All the recruited patients were advised to follow a healthy diet, rich in fruits and vegetables and poor in fats and carbohydrates (1200 Cal/day) during the study and a qualitative compliance was assessed by an interview. The patients were seen every seven days during the study. Serum aspartate aminotransferase, alanine aminotransferase, creatine kinase, blood urea nitrogen and creatinine and blood cell counts were measured before and after therapy to monitor for possible side effects.

### 2.4. Blood Sampling and Measurements

Blood samples were collected after overnight fasting at the beginning and at the end of study. The serum was collected from the samples left to clot for about 30 min and then centrifuged at 750 g for 10 min. Sera were aliquoted and frozen at −80°C until measurement. 

TotChol, LDL-C, HDL-C, TG and glucose levels in serum samples of patients were determined by commercial colorimetric and enzymatic assay kits (BioSystems S.A., Barcelona, Spain). Serum concentrations of insulin, leptin, adiponectin and ghrelin was determined by commercially available ELISA kits (Millipore, Merck S.p.a., Milano, Italy). The sensitivity of detection of leptin were 0.78–100 ng/mL, 1.5–100 μg/mL for adiponectin and 50–5000 pg/mL for ghrelin.

Weight and height were measured according to standard procedures. BMI were calculated as weight in kilograms divided by height in meters squared. The approximate insulin resistance (IR) was calculated by HOMA-IR using the following formula: glucose(mmol/L) × insulin (µU/mL))/22.5 [17]. AIP was calculated as log value of the ratio between TG and HDL-C concentrations expressed in mmol/L. 

### 2.5. Statistical Analyses

Statistical analyses were performed using GraphPad Prism version 8.0.0 for Windows, (GraphPad Software, San Diego, CA, USA). Data were expressed as a mean ± standard deviation (SD) or as a median ± SD, as indicated. Bartlett’s test was performed on each set of data to ensure that variance of the sets was homogenous. In case of homogenous set of data, one-way ANOVA with Bonferroni’s multiple comparison test was performed as appropriate. In case of heterogenous data, Kruskal–Wallis followed by Dunn’s tests were carried out. For groups comparison of categorical values, such as patient sex, Pearson’s chi-square test was performed with Statistica software (Stat Soft, Tulsa, OK, USA). 

## 3. Results

Fifty-two subjects met the inclusion criteria and were assigned to three experimental groups BPE-C high (*n* = 18), BPE-C low (*n* = 17) or placebo (*n* = 17). Forty-five subjects completed the trial. The total of seven subjects in the three groups did not complete the study due to lack of compliance. The number of drop-outs was not different between the study groups. BPE-C was also well tolerated during the study. However, similarly to our previous report [20] regarding pharmacological effects of BPF^TM^, there were few reports of moderate gastric pyrosis in BPE-C low (*n* = 2) and BPE-C high (*n* = 1) groups. Headache (*n* = 2) and constipation (*n* = 1) were reported adverse events in the placebo group. However, none of the patients taking BPE-C interrupted the treatment due to the above adverse effects. 

**Figure 1:**
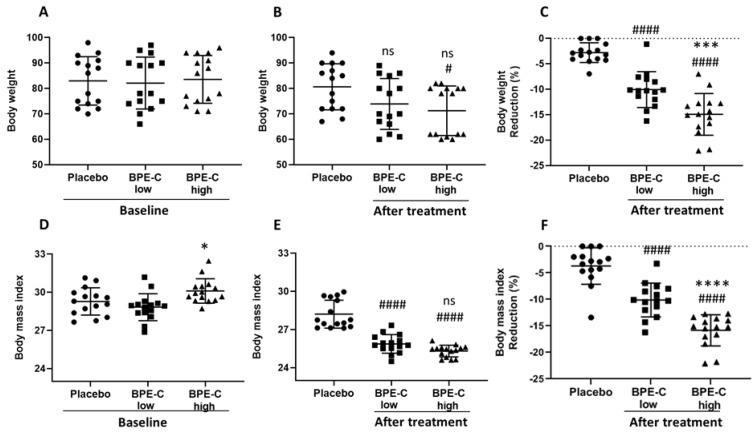
BPE-C reduces body mass (BW) in MetS patients. BW values for baseline (**A**) and (**B**) after 90-days intervention on MetS patients assigned to three experimental groups (Placebo, BPE-C low and high). (**C**) percent reduction calculated for B. BMI values for baseline (**D**) and (**E**) after 90 days as in B. (**F**) percent reduction calculated for E. The scatter plots represent 15 patients for each group. Horizontal lines and vertical bars represent the mean ± SD, respectively. Statistical analysis was performed by ANOVA followed by Bonferroni’s pos-hoc test. The analysis revealed significant differences compared to Placebo at # *p* ≤ 0.05 or #### *p* ≤ 0.0001 and between BPE-C treatment groups at * *p* ≤ 0.05, *** *p* ≤ 0.001 or **** *p* ≤ 0.0001.

**Figure 2:**
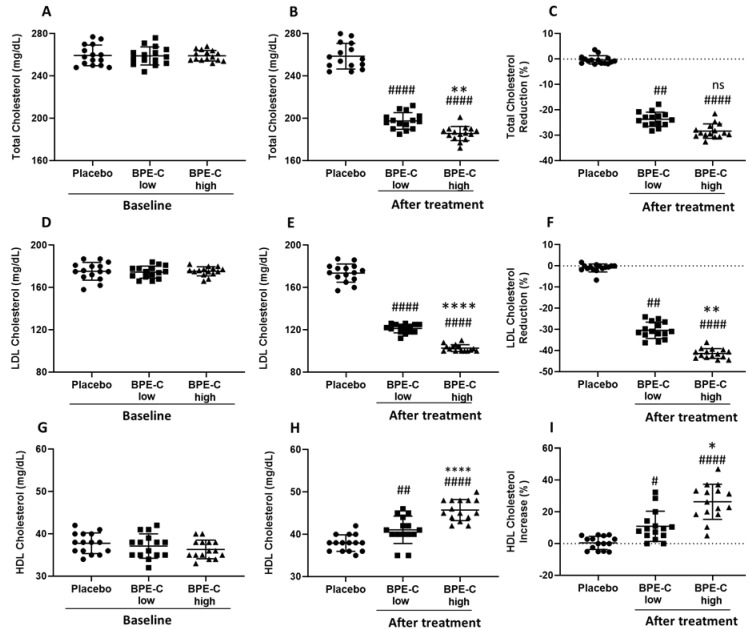
BPE-C reduces cholesterol levels in MetS patients. (**A**) Blood total cholesterol (TotChol) before (baseline) A) and (**B**) after 90-days intervention on MetS patients assigned to three experimental groups (Placebo, BPE-C low and high). (**C**) percent reduction calculated for B. (**D**) LDL-C levels for baseline and (**E**) after treatment as in B. (**F**) percent reduction calculated for E. (**G**) HDL-C levels for baseline and (**H**) after treatment as in B. (**I**) percent change calculated for H. The scatter plots represent 15 participants for each group. Statistical differences were analyzed by ANOVA (A, B, D, E, G and H) or by Kruskal–Wallis (C, F and I) followed by Bonferroni’s or Dunn’s post-hoc tests, respectively. The analysis revealed significant differences compared to Placebo at # *p* ≤ 0.05; ## *p* ≤ 0.01 or #### *p* ≤ 0.0001 and between BPE-C treatment groups at * *p* ≤ 0.05; ** *p* ≤ 0.01 or **** *p* ≤ 0.0001. Horizontal lines represent the mean ± SD.

**Figure 3:**
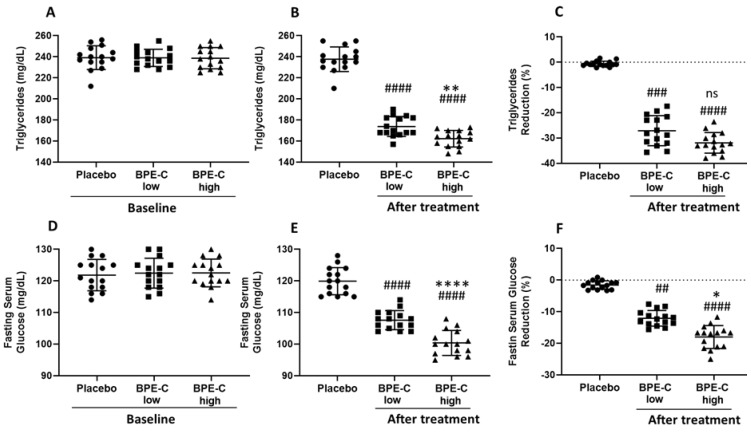
BPE-C reduces triglycerides and fasting serum glucose levels in MetS patients. (**A**) TG levels before (baseline) A) and (**B**) after 90-days intervention on MetS patients assigned to three experimental groups (Placebo, BPE-C low and high). (**C**) percent reduction calculated for B. (**D**) Baseline fasting serum glucose levels and (**E**) after 90 days as in B. (**F**) percent reduction calculated for E. Statistical differences were analyzed by ANOVA (A, B, D and E) or Kruskal–Wallis (C and F) tests followed by Bonferroni’s or Dunn’s post-hoc tests, respectively. The analysis revealed significant differences in BPE-C treatment groups when compared to Placebo at ## *p* ≤ 0.01; ### *p* ≤ 0.001 or #### *p* ≤ 0.0001 and between BPE-C treatment groups at * *p* ≤ 0.05; ** *p* ≤ 0.01 or **** *p* ≤ 0.0001. Horizontal lines represent the mean ± SD.

BPE-C and placebo groups were comparable at baseline with respect to age, sex, smoking habits (Table 1), and with respect to all study parameters: BW (Figure 1A), BMI (Figure 1C) and serum levels of TotChol (Figure 2A), LDL-C (Figure 2C), HDL-C (Figure 2E), TG (Figure 3A), and glucose (Figure 3C). It was also true when treatment groups where compared for baseline parameters, except for BMI that was significantly higher in BPE-C high when compared to BPE-C low group (Figure 1C). This baseline difference should not influence significantly BMI reduction parameter, which is calculated as a difference between BMI before and BMI after the treatment. 

After 90 days of treatment all analyzed body and serum parameters were significantly changed in BPE-C high groups, in contrast to the placebo group and in several cases also in BPE-C low groups. Notably, BW decreased significantly only in BPE-C high group (Figure 1B), but when expressed as BMI the change was significant for both low and high BPE-C groups (Figure 1E). Moreover, when compared to the baseline, BW decreased significantly by 10% and 14.8% (Figure 1C) and BMI by 10% and 15.9% (Figure 1F) in BPE-C low and high treatment groups, respectively. 

The effect of BPE-C supplementation was particularly striking and significant in case of all routinely measured cholesterol parameters, which appeared strongly decreased as in case of TotChol and LDL-C (Figure 2B,E) or elevated when compared to Placebo as in case of HDL-C (Figure 2H). The response to BPE-C was also strongly dose-dependent with *p* ≤ 0.01 for all three cholesterol parameters (Figure 2B,E,H). Moreover, when compared to baseline TotChol changed significantly by −23.7% and −28.4% (Figure 2C) and LDL-C by −30.4% and −41.4% (Figure 2F). This was associated with a marked increase in HDL-C by 10.9% and 26.4% in BPE-C low and high treatment groups, respectively (Figure 2I). 

Patients’ TG also responded strongly and dose-dependently to BPE-C low and high doses when compared to Placebo (Figure 3B) with mean reductions calculated from the baseline values such as 27.1% and 31.9%, respectively (Figure 3C). Finally, fasting serum glucose was significantly decreased after BPE-C intervention, when compared to Placebo in both treatment groups (Figure 3B). In particular, the mean changes, compared to the baseline glucose, were −12.1% and −18% in low and high treatment groups, respectively (Figure 3F). In addition, the data supported a direct and significant dose-response relationship with respect to low and high doses of BPE-C in case of TG and glucose endpoint values (Figure 3B,E). 

Finally, when “percent changes from baseline” data sets were subjected to statistical analysis we observed significant differences for BW and BMI (Figure 1C,F), LDL-C, HDL-C (Figure 2F,I) and glucose (Figure 3D) between BPE-C high and low treatment groups. In all other comparisons of the treatment groups a clear tendency to dose-dependence was observed, statistically significant according to Bonferroni’s, but not to Dunn’s multiple comparisons tests.

**Figure 4:**
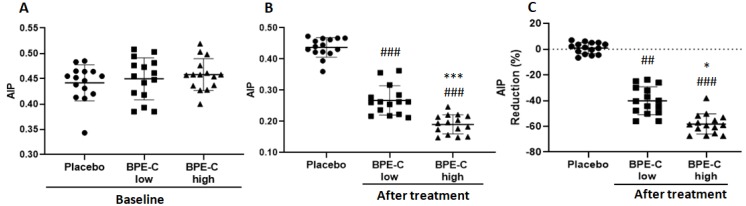
BPE-C reduces atherogenic index of plasma (AIP) in MetS patients. (**A**) AIP before treatment and (**B**) after 90-days treatment with BPE-C or placebo. (**C**) Percent reduction of AIP after treatment. Statistical differences were analyzed by ANOVA test followed by Bonferroni’s post-hoc test (A and B) or Kruskal–Wallis test followed by Dunn’s test(C). Horizontal bars show the mean of 15 patients ± SD. The analysis revealed a significant AIP decrease after the treatment with BPE-C when compared to Placebo at ## *p* ≤ 0.01 or ### *p* ≤ 0.001 for both doses of BPE-C. The response to BPE-C was dose-dependent at *** *p* ≤ 0.001 (B) or at * *p* ≤ 0.05, when % reductions were compared (C).

Next, AIP was calculated to evaluate the risk of atherogenesis and its complications after the treatment with BPE-C. The analysis revealed a significant AIP reduction (*p* ≤ 0.001), noticeable in both low and high dose groups. In particular, the starting AIP mean values 0.45 and 0.46 ± 0.04 (Figure 4A) were reduced to 0.19 ± 0.03 and 0.27 ± 0.05 in patients treated with high and low BPE-C dose, respectively (Figure 4B). In addition, the difference between low and high BPE-C groups was statistically significant (Figure 4B,C). 

**Figure 5:**
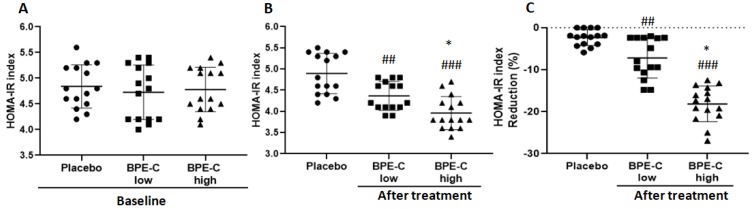
BPE-C improves insulin sensitivity by a significant reduction of HOMA-IR index in MetS patients. (**A**) HOMA-IR before treatment and (**B**) after 90-days treatment with BPE-C or placebo. (**C**) Percent change of HOMA-IR with respect to baseline values. Statistical differences were analyzed by ANOVA test followed by Bonferroni’s post-hoc test (A and B) and Kruskal–Wallis test followed by Dunn’s test (C). Horizontal bars show the mean of 15 patients ± SD. The analysis revealed a highly significant HOMA-IR decrease after the treatment with BPE-C at ## *p* ≤ 0.01 or ### *p* ≤ 0.001 for low and high doses, respectively. The response to BPE-C was dose-dependent at * *p* ≤ 0.05.

Next, we checked if the substantial reduction of hyperglycemia correlated with the improvement in insulin sensitivity in the treatment groups, approximated as reduced insulin resistance (HOMA-IR index). Between-group comparison revealed a highly significant and dose-dependent decrease of HOMA-IR in the BPE-C low and high groups compared to Placebo group (Figure 5B), which corresponded to significant mean changes by −7.2% and −18.1%, with respect to the baseline values in low and high dose groups, respectively (Figure 5C).

As expected, a significant reduction was also observed in serum insulin concentrations (*p* < 0.001) in BPE-C high patients (Table 2). Nevertheless, the response, was not significant in BPE-C low when compared to Placebo group (Table 2). 

Next, we analyzed the levels of energy balance hormones regulating fat mass and food intake. There was also no significant difference between BPE-C groups and placebo group in terms of baseline serum adiponectin and leptin, as well as for insulin and ghrelin levels (*p* > 0.05, Table 2). Posttreatment analysis revealed a significant decrease of serum leptin concentrations in both BPE-C groups (*p* < 0.05) compared to the baseline and to the placebo groups (Table 2). Likewise, the serum ghrelin was significantly reduced in the BPE-C high and low versus placebo group (*p* < 0.001) (Table 2). Finally, there was an increase in serum adiponectin concentrations in both BPE-C low and high treatment groups, which was statistically significant even in BPE-C low group compared to placebo group (*p* = 0.003) (Table 2).

## 4. Discussion

The findings of this randomized, placebo-controlled trial suggest a significant amelioration of dyslipidemia and insulin sensitivity after 90 days of BPE-C supplementation to a group of MetS patients characterized by an elevated AIP (over 0.34) and moderate hyperglycemia (up to 130 mg/L). Compared to previous clinical studies performed with BPF on a larger group MetS patients, the supplementation of BPE-C yielded similar results with respect to the reduction of TotChol, LDL-C and TG and increase of HDL-C [20]. In particular. they were slightly better for cholesterol parameters and weaker for TG. For example, a potent mean decrease by 41.4 ± 2.3% in LDL-C levels were recorded in BPE-C high group, which was a stronger response than in the previous study, in BPF high dose group (mean −36%). This might be due to a longer treatment time (90 vs. 30 days), 30% larger dose of BPE-C used in the present study as well as a specific and more homogenous profile of patients recruited for the present study. For the same reason, it is difficult to evaluate the impact of the new formulation of BPE-C with respect to the standard BPF used in the previous study [20]. Nevertheless, these data confirm that BPE-C is an efficient remedy against dyslipidemia in MetS patients with moderately elevated glycemia. 

The severity of dyslipidemia, such as high TG levels and low HDL-C, correlating with small size of LDL particles, determine high AIP and thus atherogenic risk. The value of AIP as a predictive biomarker for atherosclerosis and coronary artery disease has been confirmed in several recent studies on men and post-menopausal women [6,7,8,30,31]. The presented here data indicate a strong reduction of AIP to mean values 0.19 ± 0.03 and 0.27 ± 0.05 in patients treated with high and low BPE-C dose, respectively. Since AIP below 0.21 is considered to correlate with a low cardiovascular risk [6] we can conclude that the high dose of BPE-C is sufficient to switch the patients from high to low risk of atherosclerosis and related cardiovascular complications. 

Another important finding of this study is the dose-dependent reduction of BW and BMI by 10% to 16% in patients receiving low and high dose of BPE-C for 90 days, respectively. This is the first clinical trial demonstrating a significant BW loss with bergamot derived nutraceuticals on MetS patients. The previous clinical studies testing pharmacological effects of BPF either did not examine BW/BMI changes [19,20,21,23,24] or reported not significant BMI reduction after 120 days of BPF (1300 mg daily) supplementation [32] or found a positive preventive effect of BPF supplementation on second-generation antipsychotic drugs-induced weight gain in psychiatric patients [33]. However, the latter findings were not confirmed in a larger study [34]. In pre-clinical studies in rats, BPF effect on body mass was reported in the prevention-type studies [22,35], but not in the parallel intervention-type study on obese rats with CAF diet-induced NASH [17]. In addition, the prevention-type studies, demonstrated only a modest 8% reduction in BW gain upon 14 weeks treatment with BPF, probably due to a very aggressive type of obesogenic diet used to induce MetS with NASH, which is the CAF diet in rats [22,35]. 

Lack of a significant changes in BMI in clinical and intervention-type preclinical studies with flavonoid-only supplementation is not surprising. In fact, only one study out of 28 reviewed studies addressing the effects of dietary polyphenols on BW reported a modest weight loss after very long application of Mediterranean diet (two years) to MetS patients [16,36]. In another review, 10 out 34 papers reported on a modest weight loss induced by polyphenol extracts, in most of the cases derived from green tea [37].

Thus, dietary polyphenols rarely improve BMI parameters when applied to patients as purified polyphenol extracts. In contrast, many clinical and preclinical studies with pectins and other dietary fibers report on moderate, but significant BW loss in parallel with other health benefits related to pectin consumption [26]. Pectins usually limit BW gain by reducing food intake in rodents [28,38,39]. In humans, pectins lessen the appetite and their consumption is inversely associated with BW gain [40]. These observations indicate that body-weight loss effects of the reported here clinical study can be attributed to pectin enrichment. However, we cannot exclude that other components of bergamot juice present in BPE-C may also contribute to BW loss. 

The protective effects against diet–induced obesity are largely attributed to pectin fermentation by gut bacteria and the uptake of metabolites, such as short chain fatty acids (SCFAs) [41,42]. For example, SCFA metabolism in the liver regulates glucose and lipid metabolism and energy homeostasis [43]. A recent study demonstrates that several different types of dietary fibers reduce BW in laboratory animals and largely different gut bacteria profiles can contribute to beneficial effects of dietary fibers [28], while other studies suggest that probiotic effect of pectins depends not only on their chemical and physical characteristics [44], but also on their source. 

Energy homeostasis and, in particular body fat and food intake, as well as BMI are regulated by a complex interplay of blood hormones such a as adiponectin, leptin and ghrelin. Accordingly, the effects on BMI observed in this study were mirrored by a significant reduction in leptin levels in both treatment groups and significant upregulation of adiponectin levels. These changes likely reflect a possible reduction in body fat in patients at the end of the study.

Concurrent with a decrease in circulating leptin, plasma ghrelin concentrations increase following weight loss from an energy-restricted diet [14]. However, ghrelin levels may also decrease reflecting reduced appetite and food intake [13]. In fact, the patients in this trial experienced less appetite and they had significantly lower levels of circulating ghrelin after 90 days of BPE-C treatment. Similar observations were reported in a recent curcumin study. Daily curcumin and piperine supplementation for 12 weeks, increased adiponectin and reduced leptin and ghrelin levels, although the mean reduction in ghrelin was not significant in this study [45]. 

## 5. Conclusions

In conclusion, the present human study reports on a powerful reduction of AIP to a low risk value in a selected sub-group of MetS patients after 90 days treatment with BPE-C. It also provides the first evidence that bergamot juice-derived food supplements enriched with pectins and vitamin C, such as BPE-C significantly stimulate weight loss, improve insulin sensitivity and reduce circulating insulin, leptin, and ghrelin levels, while increasing significantly the levels of cardioprotective adiponectin. This study also confirms previously reported robust improvement of dyslipidemia, i.e, reduction of TG, TotChol and LDL-C by daily supplementation of bergamot flavonoids to the daily diet. Future studies should be undertaken to ascertain the impact of pectin and ascorbic acid, as well as other components of bergamot juice in this novel nutraceutical formulation on the observed beneficial effects.

## Figures and Tables

**Table 1:** The baseline characteristics of patients.

Variables	Placebo (*n* = 15)	BPE-C Low (*n* = 15)	BPE-C High (*n* = 15)	*p* Value (Placebo vs. BPE-C low)	*p* Value (Placebo vs. BPE-C high)	*p* Value (BPE-C low vs. high)
Age (years)	55.6 ± 7.4	59 ± 7.6	56.1 ± 10.9	0.889	≥0.999	≥0.999
Weight (kg)	83.4 ± 9.5	82.5 ± 10	84.6 ± 9.7	0.443	0.661	≥0.999
Sex (M/F)	8/7	7/8	9/6	0.715	0.713	0.464
Smokers (Y/N)	0/15	0/15	0/15	-	-	-

Age and BW data sets were analyzed by one-way ANOVA and Bonferroni’s post-hoc test. ***p*** values for sex distribution in three experimental groups were calculated using Pearson’s chi square test. High ***p*** values confirm that the three patient groups were homogenously randomized.

**Table 2:** Plasma levels of energy balance hormones and insulin, before (baseline) and after 90 days intervention on MetS patients assigned to three experimental groups (Placebo, BPE-C low and high).

Experimental Groups	Biomarker			
	Insulin (IU/L)	Leptin (ng/mL)	Ghrelin (pg/mL)	Adiponectin (mg/mL)
Baseline Placebo	20 ± 1.6	22.1 ± 1.1	619 ± 24	19.5 ± 1.3
Baseline BPE-C low	20 ± 1.5	22.6 ± 1.4	623 ± 19	19.3 ± 1.5
Baseline BPE-C high	19.6 ± 2.4	22 ± 2.3	624 ± 38	19.3 ± 1.4
Placebo	19.5 ± 2.6	22.3 ± 1.3	620 ± 22	18.6 ± 2.4
BPE-C low	16.6 ± 1.4	19.3 ± 2.0	581 ± 22	22.9 ± 2.5
BPE-C high	14.2 ± 1.2	17.3 ± 0.4	530 ± 21	23.5 ± 1.4
	*p* values			
Placebo vs. BPE-C low	0.067 ^KW^	**0.013** ^KW^	**<0.001**	**0.003**
Placebo vs. BPE-C high	**<0.001** ^KW^	**<0.001** ^KW^	**<0.001**	**<0.001**
BPE-C high vs. BPE-C low	**0.007** ^KW^	**0.046** ^KW^	**<0.001**	0.078
Change after treatment (%)				
Placebo	−2,5 ± 6.7	−0.5 ± 4	0.3 ± 2	−4.6 ± 11.6
BPE-C low	−16.5 ± 6.5	−13.9 ± 11	−6.6 ± 2.4	18.1 ± 12.8
BPE-C high	−26.4 ± 10.7	−20.5 ± 8.9	−14.9 ± 2.9	22.3 ± 9.6
	*p* values			
Placebo vs. BPE-C low	**0.001**	**0.002** ^KW^	<**0.001**	**<0.001**
Placebo vs. BPE-C high	**<0.001**	**<0.001** ^KW^	**<0.001**	**<0.001**
BPE-C high vs. BPE-C low	**0.006**	0.490 ^KW^	**0.007**	0.942

Statistical analysis was performed by ANOVA and Bonferroni’s post-hoc test (where not specified) or by Kruskal–Wallis followed by Dunn’s post-hoc, where indicated by KW. The mean ± SD out of 15 patients is shown for each parameter. Statistically significant *p* values (*p* < 0.05) are in bold.
